# Gamified Crowdsourcing as a Novel Approach to Lung Ultrasound Data Set Labeling: Prospective Analysis

**DOI:** 10.2196/51397

**Published:** 2024-07-04

**Authors:** Nicole M Duggan, Mike Jin, Maria Alejandra Duran Mendicuti, Stephen Hallisey, Denie Bernier, Lauren A Selame, Ameneh Asgari-Targhi, Chanel E Fischetti, Ruben Lucassen, Anthony E Samir, Erik Duhaime, Tina Kapur, Andrew J Goldsmith

**Affiliations:** 1 Department of Emergency Medicine Brigham and Women's Hospital Harvard Medical School Boston, MA United States; 2 Centaur Labs Boston, MA United States; 3 Department of Radiology Brigham and Women's Hospital Harvard Medical School Boston, MA United States; 4 Department of Biomedical Engineering Eindhoven University of Technology Eindhoven Netherlands; 5 Department of Radiology Massachusetts General Hospital Harvard Medical School Boston, MA United States; 6 Department of Emergency Medicine Lahey Hospital University of Massachusetts Medical School Burlington, MA United States

**Keywords:** crowdsource, crowdsourced, crowdsourcing, machine learning, artificial intelligence, point-of-care ultrasound, POCUS, lung ultrasound, B-lines, gamification, gamify, gamified, label, labels, labeling, classification, lung, pulmonary, respiratory, ultrasound, imaging, medical image, diagnostic, diagnose, diagnosis, data science

## Abstract

**Background:**

Machine learning (ML) models can yield faster and more accurate medical diagnoses; however, developing ML models is limited by a lack of high-quality labeled training data. Crowdsourced labeling is a potential solution but can be constrained by concerns about label quality.

**Objective:**

This study aims to examine whether a gamified crowdsourcing platform with continuous performance assessment, user feedback, and performance-based incentives could produce expert-quality labels on medical imaging data.

**Methods:**

In this diagnostic comparison study, 2384 lung ultrasound clips were retrospectively collected from 203 emergency department patients. A total of 6 lung ultrasound experts classified 393 of these clips as having no B-lines, one or more discrete B-lines, or confluent B-lines to create 2 sets of reference standard data sets (195 training clips and 198 test clips). Sets were respectively used to (1) train users on a gamified crowdsourcing platform and (2) compare the concordance of the resulting crowd labels to the concordance of individual experts to reference standards. Crowd opinions were sourced from DiagnosUs (Centaur Labs) iOS app users over 8 days, filtered based on past performance, aggregated using majority rule, and analyzed for label concordance compared with a hold-out test set of expert-labeled clips. The primary outcome was comparing the labeling concordance of collated crowd opinions to trained experts in classifying B-lines on lung ultrasound clips.

**Results:**

Our clinical data set included patients with a mean age of 60.0 (SD 19.0) years; 105 (51.7%) patients were female and 114 (56.1%) patients were White. Over the 195 training clips, the expert-consensus label distribution was 114 (58%) no B-lines, 56 (29%) discrete B-lines, and 25 (13%) confluent B-lines. Over the 198 test clips, expert-consensus label distribution was 138 (70%) no B-lines, 36 (18%) discrete B-lines, and 24 (12%) confluent B-lines. In total, 99,238 opinions were collected from 426 unique users. On a test set of 198 clips, the mean labeling concordance of individual experts relative to the reference standard was 85.0% (SE 2.0), compared with 87.9% crowdsourced label concordance (*P*=.15). When individual experts’ opinions were compared with reference standard labels created by majority vote excluding their own opinion, crowd concordance was higher than the mean concordance of individual experts to reference standards (87.4% vs 80.8%, SE 1.6 for expert concordance; *P*<.001). Clips with discrete B-lines had the most disagreement from both the crowd consensus and individual experts with the expert consensus. Using randomly sampled subsets of crowd opinions, 7 quality-filtered opinions were sufficient to achieve near the maximum crowd concordance.

**Conclusions:**

Crowdsourced labels for B-line classification on lung ultrasound clips via a gamified approach achieved expert-level accuracy. This suggests a strategic role for gamified crowdsourcing in efficiently generating labeled image data sets for training ML systems.

## Introduction

### Background

Machine learning (ML) models can improve medical diagnostic concordance and streamline health care processes [1]. This is particularly true when applied to medical image analysis. ML models require large-scale labeled data sets for model training [2]. Widespread ML tool development is limited by the need to acquire high-quality labels given the associated expert time and cost [3-7].

Combining opinions from multiple individuals on a given task can produce more accurate interpretations than from a single individual [8]. Crowdsourcing, the process of collecting large numbers of unskilled opinions, can improve efficiency, lower costs, and offer high quality in repetitive task completion [8,9]. Crowdsourced approaches to data set labeling are growing in popularity, and beneficial effects of crowdsourcing have been demonstrated in health care–related tasks including biomedical imaging analysis [10-14].

Using crowdsourcing for biomedical image labeling is challenged by the complexity of the tasks and the need to ensure label quality control. The user interface design for collecting crowd opinions and the metrics used for assessing opinion quality is key to successful results. Gamification, the persuasive system design that uses game-like tasks to engage participants competitively for rewards, can both encourage crowd participation and improve performance accuracy by selectively rewarding top users [15-18]. Combining crowdsourcing with gamification can become a performance measurement tool for identifying top crowd labelers [18-21].

Point-of-care ultrasound (POCUS) is a dynamic medical imaging technique used at patients’ bedside to make accurate, real-time diagnoses [22-24]. Though POCUS has significant value in health care settings, advanced training is required to accurately apply this tool to clinical care [25]. As such, ML models that automate POCUS image interpretation hold exceptional potential clinical value. In lung POCUS, B-lines are hyperechoic linear artifacts that extend from the pleural line and appear dynamically with the respiratory cycle [23]. B-lines are known markers of pulmonary congestion and their presence, quantity, and thickness (discrete vs confluent B-lines) correlate with pathological severity of conditions such as congestive heart failure exacerbations, pneumonia, or inflammatory lung disease [23,26,27]. Early ML models for lung POCUS have shown promise in identifying and quantifying the presence of B-lines [28-33]. However, existing models have limited accuracy and generalizability due in part to the lack of large-scale, high-quality, labeled image databases for model training. Gamified crowdsourcing has not previously been applied to annotate lung POCUS clips or ultrasound imaging data overall.

### Objectives

We examined whether a gamified crowdsourcing approach with inbuilt quality control measures can classify lung POCUS clips for presence and type of B-lines at comparable concordance to trained ultrasound experts. Our secondary objectives were to measure (1) the number of crowd opinions required to achieve maximal concordance, and (2) learning curves for expert and crowd individuals over time.

## Methods

### Study Design and Setting

This was a prospective analysis performed using retrospectively collected lung POCUS clips. All lung POCUS examinations performed in an academic tertiary care emergency department (ED) between March 1, 2020, and February 28, 2022, were retrospectively queried via the eHealth record.

### Data Set Curation

In total, 2391 POCUS clips were downloaded in Digital Imaging and Communications in Medicine format from the hospital Picture Archiving and Communication System, and 6-second clips were acquired at frame rates between 15 and 46 Hz. Digital Imaging and Communications in Medicine files were converted to MP4 format using an open-source medical image viewer and subsequently deidentified using a software package [34,35].

**Figure 1:**
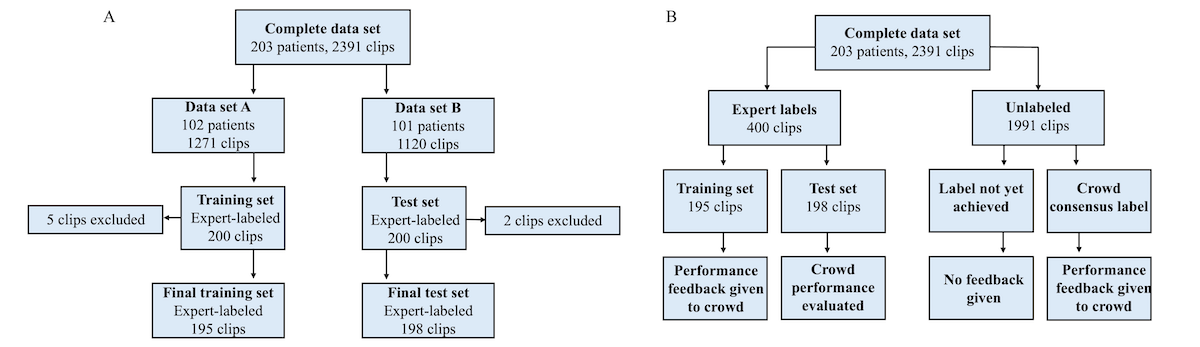
Data set processing flow diagram. (A) Complete data set partitioning. All 2391-lung point-of-care ultrasound (POCUS) clips from the complete data set were divided evenly by the patient into data set A and data set B. A total of 200 clips from each sub-data set were randomly selected to create a training set and test set, respectively. A total of 5 clips from the training set and 2 clips from the test set were excluded for having no lung visible in the clip prior to deploying these sets to the crowd. (B) Lung POCUS clip labeling workflow. Of the complete data set, 400 clips (200 from data set A and 200 from data set B) were separated to become the training set, used for giving performance feedback to the crowd, and the test set, used to evaluate crowd performance and concordance. The remaining clips from the complete data set were not labeled by experts but instead were available to achieve a crowd-consensus label. Crowd users were given additional performance feedback on clips that had previously achieved a crowd-consensus label.

The 2391 clips were randomly divided by patient into 2 sets: data set A (102 patients, 1271 clips) and data set B (101 patients, 1120 clips). A total of 200 random clips from data set A were selected as a crowd training set, and 200 random clips from data set B were selected as a test set to evaluate crowd label quality. A total of 5 training set clips and 2 test set clips were excluded for being flagged by at least 1 expert as not containing lung (Figure 1A).

### Task Definition

**Figure 2:**
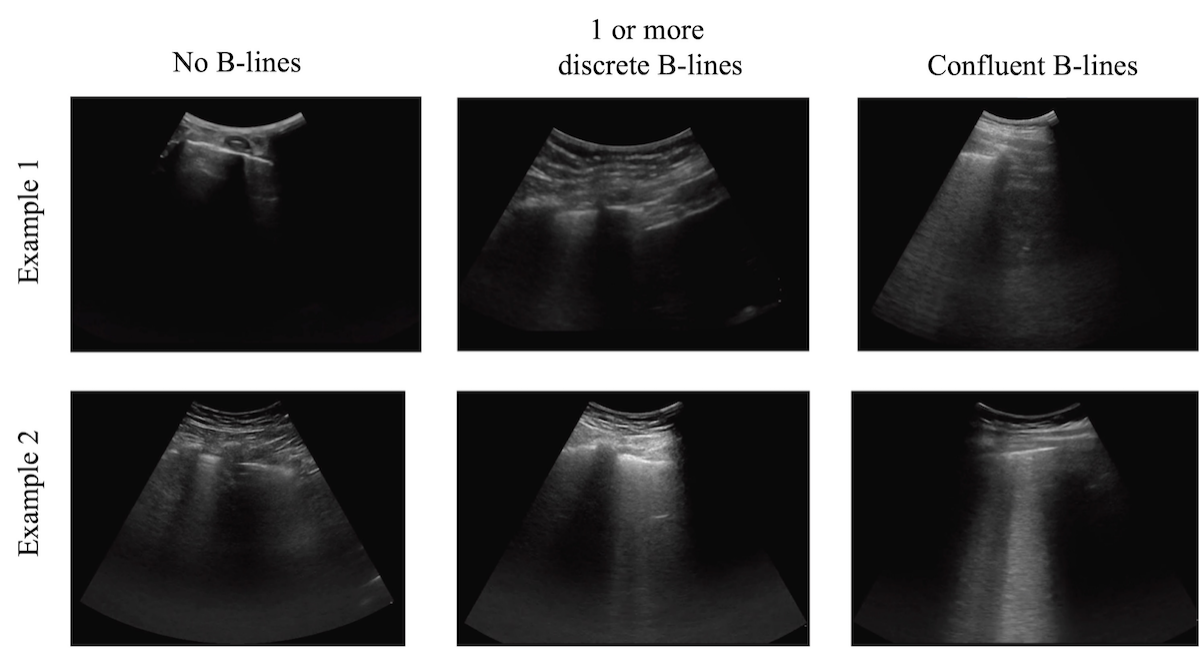
Representative frames from lung point-of-care ultrasound (POCUS) images demonstrating the 3 potential classification label categories. (1) Two lung POCUS images demonstrating no B-lines; (2) Two lung POCUS images demonstrating discrete B-lines; (3) Two lung POCUS images demonstrating confluent B-lines.

Expert and crowd users were asked to classify B-lines on lung POCUS clips into one of three classes: (1) no B-lines, (2) one or more discrete B-lines, or (3) confluent B-lines (Figure 2). Clips were classified based on the highest B-line severity present throughout the entire clip, with no B-lines < one or more discrete B-lines < confluent B-lines. Discrete B-lines were defined as hyperechoic lines originating from the pleural line, demonstrated sliding with the pleura, and extending to the bottom of the sonographic field [36,37]. Confluent B-lines were defined as hyperechoic sections originating from the pleural line, demonstrated sliding with the pleura, and had thickness along the pleura beyond that of discrete B-lines [36,37].

A total of 6 experts with advanced training in lung POCUS (4 ultrasound fellowship-trained emergency medicine physicians including 1 dual board-certified in intensive care; 1 emergency radiologist, and 1 registered diagnostic medical sonographer) were recruited. All 6 experts provided independent classification opinions for all training and test set clips via DiagnosUs, a free iOS app where users compete in medical data labeling contests to win cash prizes based on their labeling concordance. All clips could be continuously played or viewed frame-by-frame as many times as needed. Prior to participation, experts underwent a 30-minute training session familiarizing themselves with the app and ensuring agreement on the study-specific classifications.

### Reference Standard Calculations

Reference standard labels on training and test set clips were assigned using expert consensus—the majority rule of the 6 experts’ opinions, with ties broken randomly. For the single reference standard based on all 6 experts’ opinions, the average expert concordance with the reference standard was calculated as the average of the 6 individual expert concordances with the reference standard.

A collection of 6 “leave-one-out” reference standard label sets was created based on 5 experts each, 1 for each expert whose input was excluded from the majority rule consensus. This was done to measure each expert against a reference standard which their own opinion did not influence. For concordances with respect to leave-one-out reference standards, an individual expert’s concordance was the proportion of their opinions that matched their respective set of leave-one-out reference standard labels (the set of labels excluding their own opinion), and the crowd concordance was the proportion of the 6 leave-one-out reference standard labels matched by the crowd consensus label. The overall crowd concordance was the average of the per-clip crowd concordances.

### Gamified Crowdsourcing

Crowd opinions were collected using DiagnosUs via gamified contests. Crowd users included anyone in the general public with access to an iOS device who downloaded the iOS app. There were no criteria regarding the level of expertise to participate. Users voluntarily participated in labeling contests based on interest and the potential to earn rewards.

Crowd users were trained in two ways as follows: (1) optional tutorial cases with accompanying layperson explanations which they could access at any time and (2) after submitting an opinion, immediate feedback was given via revelation of the current label (ie, the reference standard label on training set clips, or the current crowd consensus label if one exists). Crowd consensus labels were computed and assigned for all clips using the majority rule of the top crowd labelers’ opinions, and were assigned to any of the 1991 initially unlabeled clips once sufficient mutual agreement was reached among the top crowd labelers for that clip (Figure 1B). Top labelers were identified via continuous performance monitoring based on their trailing concordance on clips with labels. Clips were shown to users in random order so that they could not predict which clips would impact their contest score, and any test clip could be shown to a user multiple times. Additional details regarding the mechanics of crowd opinion collection and aggregation are provided in Multimedia Appendix 1.

### Gamified Crowdsourcing Contest Mechanics

Users participating in classification contests were shown lung POCUS clips in random order. After submitting their opinion on a clip, they were either shown the correct label as feedback (on feedback clips) or a message thanking the user for their opinion (on nonfeedback clips). Feedback clips consisted of the 195 training set clips with reference standard labels, as well as any of the 1991 unlabeled clips that had acquired a crowd consensus label (Figure 1B). Feedback clips shown to the user were sampled to be equally likely to have a correct label in each of the 3 B-line classifications. Nonfeedback clips included the 198 test clips with reference standard labels, as well as any of the 1991 unlabeled clips that had yet to reach crowd consensus. Due to randomized clip ordering, users did not know which clips were feedback clips, and therefore, which clips would be used to grade their labeling performance for prizes. The live contest was run until all unlabeled clips reached crowd consensus.

### Crowd Consensus Labeling and Quality Assessment

Crowd consensus (ie, the B-line classification with the most crowd votes, with ties broken randomly) was achieved for an initially unlabeled clip when either (1) a minimum 3-vote difference between the highest and second highest-voted classification choice was achieved, or (2) 15 opinions were collected for the clip. If a user contributed multiple opinions to the same clip, only their most recent opinion was considered. Once crowd consensus was reached on an unlabeled clip, the clip acquired a crowd consensus label and became a feedback clip.

Crowd label quality was assessed by comparing how well crowd consensus labels and individual expert opinions respectively matched the reference standard labels on the test set. Both of these were measured by concordance, calculated simply as the percent of matching labels across the test set clips. In addition, we also calculated concordances with “leave-one-out” reference standards.

A crowd user’s opinion quality score (Qscore) was defined as the user’s trailing average concordance (with respect to reference standards for training clips and crowd consensus labels for initially unlabeled clips) on the last 50 feedback clips they provided an opinion on. Each user’s Qscore was dynamically tracked over time and was updated with each opinion they gave. If a user’s Qscore was less than 80% at the time they gave an opinion on an individual clip, that opinion was not considered for determining the crowd consensus for that clip and instead was discarded. Users were assigned a Qscore of 0 until they provided at least 25 opinions on feedback clips. This ensured only the highest quality crowd opinions contributed to crowd consensus labels.

### Analysis of Secondary Outcomes

To assess the minimum number of crowd opinions needed to achieve expert-level concordance, we estimated the concordance that the crowd labels would have had if we had collected fewer opinions on each clip by repeatedly sampling fewer opinions from all test clips and computing concordance with the reference standard based on the resulting crowd consensus labels. Just as crowd consensus labels were determined as described above (“Crowd Consensus Labeling and Quality Assessment”), only opinions submitted by users whose Qscore was at least 80% at the time of opinion submission were considered when sampling. For every opinion count, we used 1000 Monte Carlo samples per clip to estimate concordance.

To calculate learning curves, the concordance of individual crowd users or experts with the reference standard was computed each time the individual gave an opinion on a test set clip, based on the proportion of their last 25 opinions on test set clips that matched the reference standard. The concordance at each opinion submission was averaged across individuals (all crowd users, skilled crowd users, or all experts) to produce the learning curves. Skilled crowd users were users who submitted at least 1 opinion on at least 1 test clip when they had a trailing average concordance (with respect to reference standards for training clips and crowd consensus labels for initially unlabeled clips) of 80% or better. For this analysis, all opinions from crowd users were considered, including multiple opinions per user per clip, if applicable.

### Statistical Analysis

Analysis was performed using Python (version 3.10; Python Software Foundation) [38]. The difference between crowd concordance and average individual expert concordance was tested for significance using a paired-samples *t* test. For concordance based on the 6-expert consensus, the crowd concordance for each case was 1 if the crowd label was correct and 0 otherwise, and the average expert concordance for that case was the proportion of the 6 experts that were correct. For leave-one-out concordance based on 5-expert consensus, the crowd concordance for each case was the proportion of the 6 leave-one-out consensus labels that matched the crowd label, and the average expert concordance for that case was the proportion of the 6 experts that matched their respective leave-one-out consensus label. All mean calculations are reported as mean (SE).

### Ethical Considerations

This study protocol was approved by the Mass General Brigham institutional review board (protocol number 2021P001446). Primary lung ultrasound data set creation was considered exempt from obtaining patient consent by the institutional review board given that the data set was collected retrospectively from the medical center’s clinical image archive and all data were deidentified prior to use in this study. DiagnosUs users provided consent for their data to be used for research purposes prior to engaging in any labeling contests. No compensation was provided for participating in this study.

## Results

### Data Set Characteristics

The patients who contributed to the lung POCUS database had a mean age of 60.0 (SD 19.0) years, and 105 (51.7%) patients were female, 43 (21.2%) patients were Hispanic, 42 (20.7%) patients were Black, and 114 (56.1%) patients were White. From the ED, 122 (64%) of patients were admitted to the hospital floor, 57 (28.6%) were discharged home, 13 (6.4%) were admitted to the intensive care unit, 2 (1%) went directly to the operating room, 9 (4.4%) had an alternate disposition, and 0 (0%) expired in the ED (Table 1).

**Table 1:** Patient characteristics from the lung point-of-care ultrasound data set used for image annotation. Clips were queried retrospectively via the eHealth record from clinical lung ultrasound examinations performed for patients presenting to the emergency department between March 1, 2020, and February 28, 2022.

Characteristic		Subjects, (N=203), n (%)
**Sex**		
	Male	98 (48.3)
	Female	105 (51.7)
**Ethnicity**		
	Hispanic or Latino	43 (21.2)
	Non-Hispanic or Latino	160 (78.8)
**Race**		
	American Indian or Alaskan Native	0 (0)
	Asian	9 (4.4)
	Black or African American	42 (20.7)
	Caucasian	114 (56.2)
	Native Hawaiian or Pacific Islander	0 (0)
	Other	38 (18.7)
**Emergency department disposition**		
	Discharge home	57 (28.6)
	Floor admission	122 (64.0)
	Intensive care unit admission	13 (6.4)
	Operating room	2 (1.0)
	Other	9 (4.4)

### Opinion Collection

Experts spent an average of 1.7 (range 0.9-2.5) hours submitting opinions for all 393 training and test clips (3.9 opinions per minute on average). Over the 195 training clips, the reference standard label distribution (based on the experts’ majority opinion) was 58% (n=114) no B-lines, 29% (n=56) discrete B-lines, and 13% (n=25) confluent B-lines. Over the 198 test clips, the reference standard label distribution was 70% (n=138) no B-lines, 18% (n=36) discrete B-lines, and 12% (n=24) confluent B-lines.

In total, 99,238 crowdsourced opinions were collected from 426 unique users across all 2384 clips. The number of users contributing an opinion to each test set clip ranged from 28 to 48. Of these, 34,363 opinions from 114 unique users were eligible to be considered for crowd consensus labels based on our quality thresholds. Of the 114 users who contributed to crowd consensus labels, 53% (n=60) had prior medical experience, compared to 45% (n=190) of users overall. A total of 56,874 (57.3%) opinions were from users who reported prior medical experience, as were 22,231 (64.7%) opinions eligible for consideration for crowd consensus labels. The live contest was launched over 138 hours which reflected a mean acquisition rate of 12.0 opinions per minute. The total cash prize payout throughout the entire competition was US $1100. The maximum prize earned by an individual user was US $25.

### Label Concordance With Reference Standard

**Figure 3:**
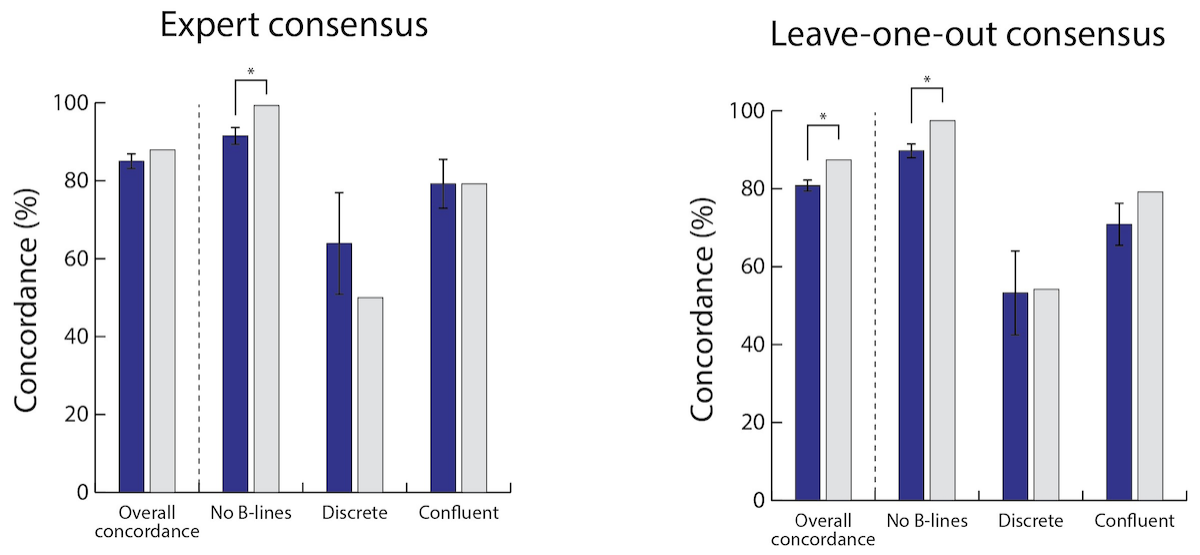
Expert and crowd label concordance with reference standards on test set clips stratified by B-line classification. (A) Expert and crowd label concordance for each B-line classification type compared with the reference standard comprised of consensus of 6 experts. Overall, on all clips combined, there was no difference in the crowd concordance of all clips compared with individual experts’ concordance (*P*=.15). For clips designated by the reference standard as having no B-lines, crowd concordance exceeded average expert concordance compared with a reference standard (*P*<.001). (B) Expert and crowd label concordance for each B-line classification type compared with the reference standard comprised of consensus of 5 experts with the opinion of the individual expert being tested left out (ie, leave-one-out reference standard). When compared with the leave-one-out reference standard, crowd concordance exceeded expert concordance both for all B-line classifications combined, as well as for clips with no B-lines. *P<.001.

The 6 experts’ concordances on the 198 test clips relative to the reference standard were 77.2%, 81.3%, 84.8%, 87.3%, 88.4%, and 90.9%, with a mean of 85.0 (SD 2.0). Comparatively, the crowd concordance on these clips was 87.9% relative to the reference standard (*P*=.15; Figure 3A). When individual expert concordances were computed against “leave-one-out” reference standards, expert concordances on the same 198 test clips were 75.8%, 77.8%, 79.8%, 81.8%, 83.3%, and 86.4% with a mean of 80.8 (SD 1.6), compared with a crowd concordance of 87.4% (*P*<.001; Figure 3B).

For clips designated by the reference standard as having no B-lines, experts had an average concordance with the reference standard of 91.5% (SD 2.3), compared with a crowd concordance of 99.3% (*P*<.001). For cases with discrete B-lines, experts had an average concordance of 63.9% (SD 13.2) compared with the crowd concordance of 50% (*P*=.09). For cases with confluent B-lines, expert average concordance (mean 79.2%, SD 6.5) and crowd concordance were both 79.2% (*P*>.99). The balanced multiclass concordance (ie, average per-class concordance) was 76.1% for the crowd and 78.2% (SD 4.0) for individual experts relative to the reference standard, and 76.9% for the crowd and 71.2% (SD 3.2) for individual experts relative to leave-one-out reference standard.

**Figure 4:**
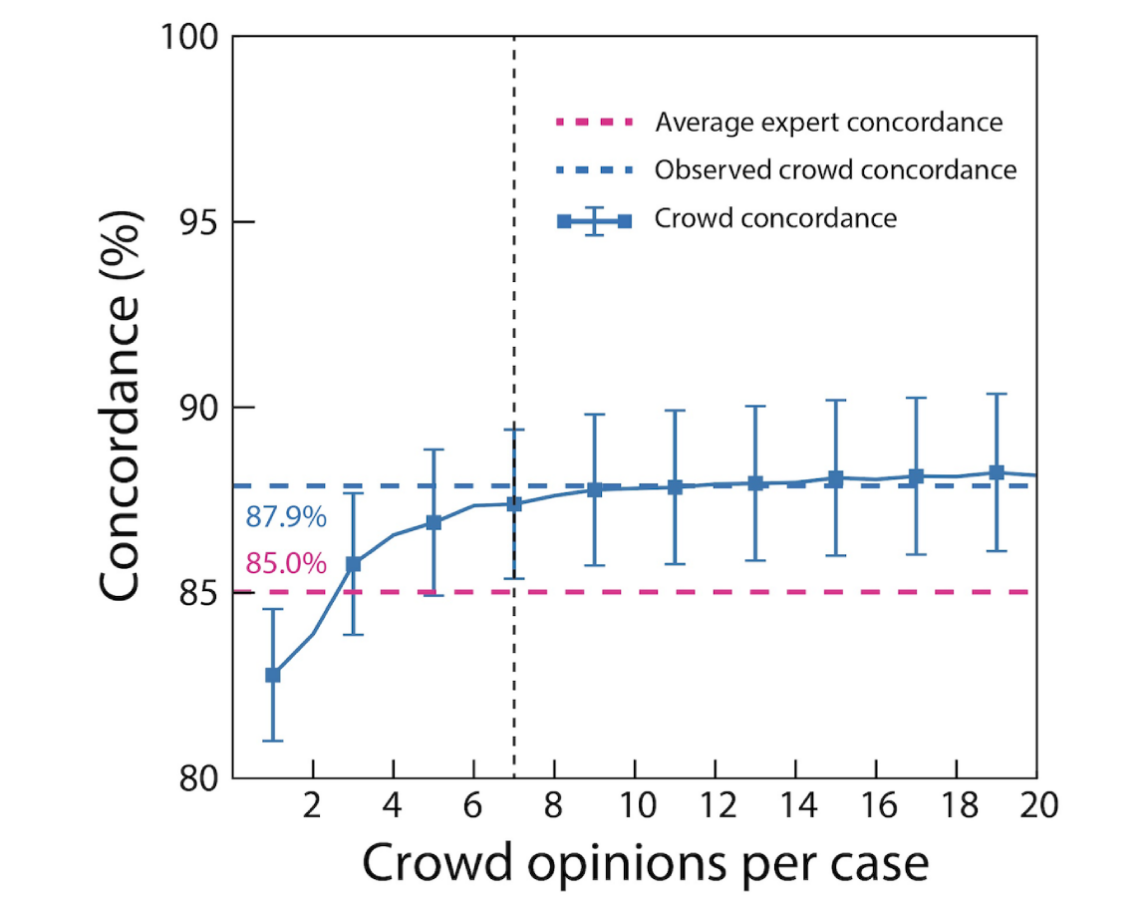
Determining the number of crowd opinions needed to maximize crowd-consensus concordance using randomly sampled subsets of collected opinions. The solid blue line indicates estimated crowd-consensus concordance as dependent on the number of crowd opinions collected. The vertical dotted line indicates that 7 crowd opinions are sufficient to achieve the desired crowd concordance, and the data suggest that further opinion collection shows diminishing marginal increases in crowd consensus concordance. Observed crowd concordance (horizontal dotted blue line) indicates the overall crowd-consensus concordance from using all crowd opinions on each clip. Average expert concordance (horizontal dotted red line) indicated the combined expert concordance. Error bars indicate the SE of the mean.

Using randomly sampled subsets of collected opinions, 7 quality-filtered opinions were sufficient to achieve near the maximum crowd concordance (Figure 4).

### Expert and Crowd Labeling Disagreements

Expert opinions were unanimous on 50.3% (n=98) of training cases and 52.5% (n=104) of test cases. There was an expert supermajority (ie, two-thirds) on 89.2% (n=174) of training cases and 87.9% (n=174) of test cases. On average, 41.2 (SD 4.1) crowd opinions contributed toward the crowd consensus label for each of the test cases. Crowd opinions were unanimous in 35.4% of cases, and a crowd supermajority existed for 85.9% (n=170) of test cases.

**Figure 5:**
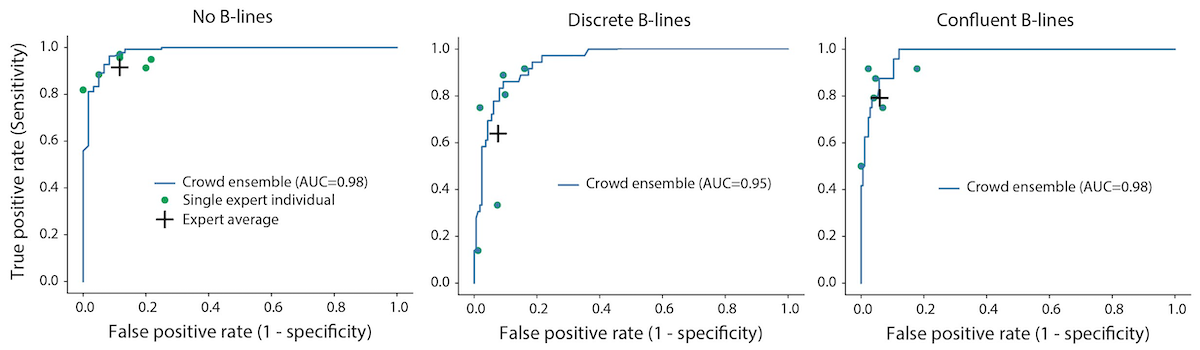
Receiver operating characteristic (ROC) curves for crowd consensus labels relative to the expert-consensus label by B-line classification using different thresholds of crowd opinion proportions to predict the expert-consensus label. ROC curves for all B-line classifications produced a high AUC. Sensitivity and specificity for individual experts are plotted as green points, and the average sensitivity and specificity of all 6 experts are shown as a black cross. (A) ROC curve for clips with no B-lines, (B) ROC curve for clips with discrete B-lines, and (C) ROC curve for clips with confluent B-lines. AUC: area under the curve.

The receiver operating characteristic curves based on using different thresholds of crowd opinion proportions to predict the expert-consensus label produced high area under the curve values for all B-line classifications: the area under the curve was 0.98 for the “No B-lines” classification, 0.95 for “discrete B-lines,” and 0.98 for “confluent B-lines” (Figure 5).

**Figure 6:**
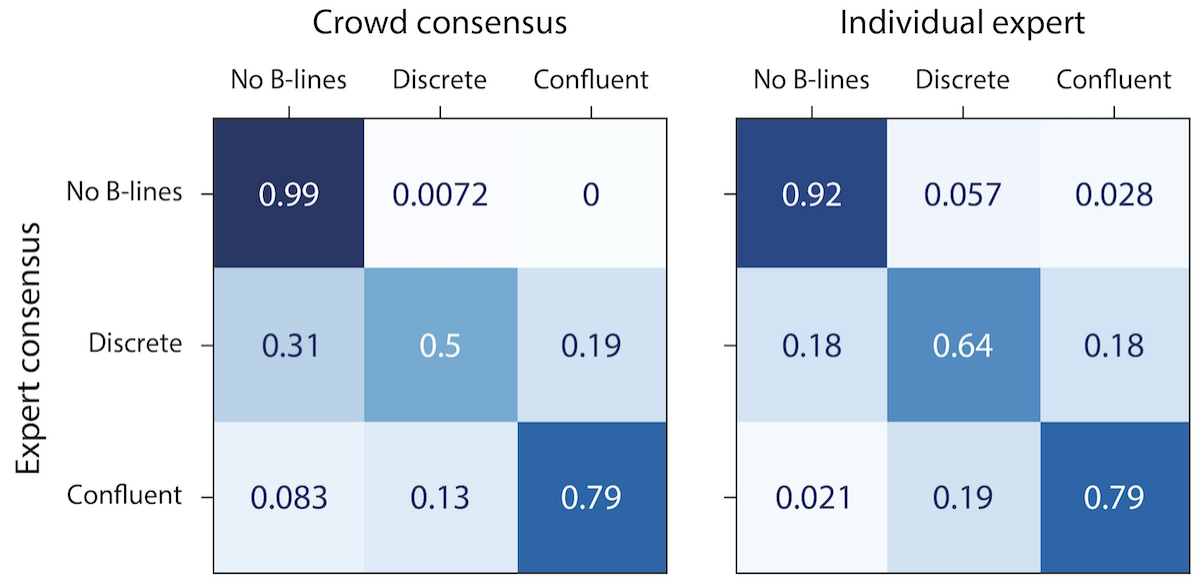
Confusion matrices showing disagreement by category between the expert consensus (A), the crowd consensus, and (B) the average of individual experts. Clips with discrete B-lines had the most disagreement from both the crowd consensus and individual experts with the expert consensus, making up 75% of cases where the crowd consensus differed from the expert consensus reference standard.

Clips with discrete B-lines had the most disagreement from both the crowd consensus and individual experts with the expert consensus, making up 75% (n=18) of cases where the crowd consensus differed from the expert consensus reference standard (Figure 6).

On a clip-by-clip basis, the level of internal crowd agreement and internal expert agreement on each test clip was significantly correlated (Pearson *r*=0.70, *P*<.001). When considering only test clips where the crowd opinions were at least 80% in agreement, the crowd consensus label concordance with the reference standard was 96%. The expert agreement was significantly higher on test clips where the crowd label matched the reference standard (89%) versus on test clips where it did not (60%; *P*<.001, Mann-Whitney *U* test).

### Expert and Crowd Learning

**Figure 7:**
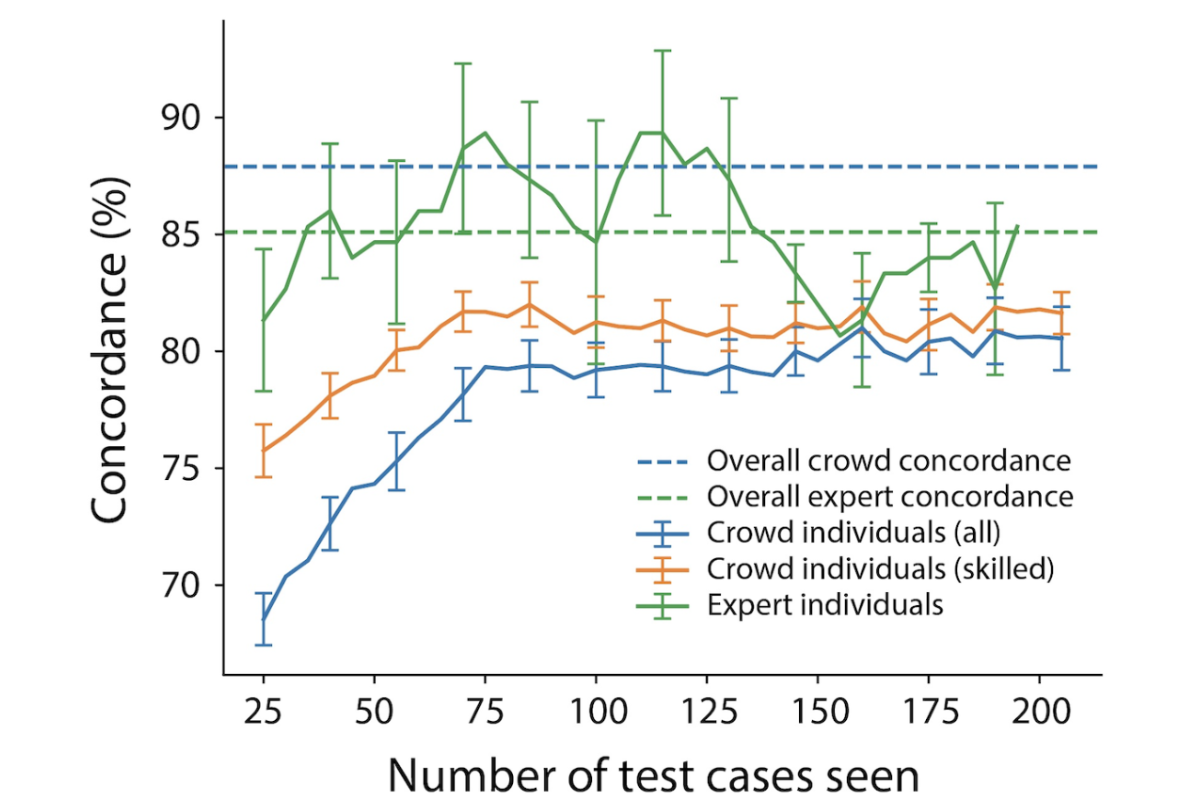
Learning curves for experts and the crowd separated into all individuals and individuals who were designated as skilled based on a user opinion quality score of >80% as measured by trailing average concordance relative to the reference standard, plotted versus the number of test set cases seen. A window of 25 cases was used to compute trailing average concordance. Individual crowd users showed improved concordance with the reference standard over time as each user gave opinions on more clips and thus received more feedback, reaching a final average concordance of 80%-81% after seeing around 75 test set clips. Error bars represent the SE of the mean.

Individual crowd users showed improved concordance with the reference standard over time as each user gave opinions on more clips and thus received more feedback. Individual crowd users reached a final average concordance of 80%-81% after seeing around 75 test set clips (Figure 7). Experts maintained a relatively constant concordance level with the reference standard regardless of the number of test cases seen. The subset of skilled crowd users (n=114) showed an intermediate effect, having a slightly higher concordance than the overall crowd at all times and a less dramatic concordance increase, but still reaching a maximum individual concordance of around 81%.

## Discussion

### Principal Findings

Our data suggest that gamified crowdsourcing can offer an efficient means of obtaining expert-quality labels for B-line classification on lung POCUS clips. While our crowdsourcing contests are open to and attract users of all skill levels from the general population, our approach of filtering highest performing opinions from the crowd allows us to collate a group of semiskilled labelers whose collective intelligence performs on par with, or in some cases outperforms, individual credentialed experts. This method offers a more efficient means of creating labels on medical imaging data than traditional expert-derived approaches. A filtering Qscore of 80% was selected in this work as this gives a reasonable balance between concordance and label acquisition speed based on preliminary data. Future studies could focus on identifying the lowest possible filtering Qscore at which crowd performance continues to outpace that of experts for a given labeling task. This may improve the efficiency of medical data labeling further and could help identify a skill threshold by which crowd input can be filtered while still achieving expert-level concordance. Individual expert accuracies for classifying B-lines ranged considerably. This finding could have 2 possible explanations. First, given that medical imaging data interpretation is often complex, it may be that a proportion of clips have inherent ambiguity that even experts disagree on. This is consistent with previously published work that shows expert interrater agreement for identifying B-lines while substantial, is often imperfect [39-42]. Further, crowd inaccuracy and internal disagreement correlated with expert internal disagreement. Thus, it is possible that clips demonstrating crowd “inaccuracy” may actually be clinically equivocal rather than reflecting poor skill by the crowd. Inherent ambiguity is likely a theme that extends across medical imaging data beyond lung POCUS and may represent a challenge for imaging database labeling overall.

Variability in expert concordance could also be attributable to variable expert baseline skill. Consistent with existing literature that uses medical experts to label POCUS images, our experts all had either fellowship-level training or advanced certification in interpreting lung ultrasound, as well as years of clinical experience [28-33]. Ground truth labeling for training POCUS-based ML models is commonly derived from a small handful of experts (typically 1-5 individuals). Thus, this work combining opinions from 6 experts (or 5 experts in the case of “leave-one-out consensus”) to form our reference standard is consistent with accepted practices. Currently, there is no established method for defining ground truth in B-line identification beyond expert opinion. Given the recent widespread adoption of lung POCUS globally and the recognized utility of B-lines as a clinical disease marker, our work highlights the critical need for clarifying how ground truth interpretation of lung POCUS and POCUS overall is defined.

Individual crowd users improved by their opinions converging toward the reference standard over the course of contest participation whereas expert concordance with the reference standard remained high throughout. This is expected as experts should not gain significant benefits beyond their baseline skill level from additional cases and thus are not expected to demonstrate a learning curve. This suggests that nonexpert users can dynamically adapt to task-specific instructions or features based on real-time feedback. Even with tutorial- and feedback-based learning, individual crowd users generally did not improve to the concordance level of the average individual expert, but the combination of crowd users was able to exceed individual expert concordance. This illustrates the “wisdom of the crowd” effect of gaining concordance from incorporating multiple opinions from users who are individually less skilled than experts to reach a concordance level equal to that of experts [43].

While commercial crowdsourcing platforms that allow users to perform discrete, repetitive tasks, such as data labeling, exist, our approach increased labeling concordance and streamlined worker skill assessment via gamification. Gamified contests are structured to ensure crowd users put forth maximal effort, and offer users the opportunity for skill enhancement and learning via immediate feedback. Contest mechanics dynamically estimate user skill levels even as they fluctuate with learning from a subset of expert-annotated clips. The combination of continual user learning and selectively filtering for the most skilled users’ opinions enhances labeling concordance. While this approach does initially require a small number of expert-quality labels for crowd training, this input is a significant improvement in expert effort over what is commonly required for data set labeling today. The novelty in this work is not through crowdsourcing alone, but rather in incorporating a gamified approach that encourages optimal user performance through a system of rewards. Using inbuilt quality control metrics in our platform, we are then able to filter the highest-performing labelers from the general crowd to curate a set of labels that have a level of concordance that exceeds that of the general participating crowd. Incorporating both gamification and quality filtering techniques into a crowdsourced task is what allows us to efficiently generate expert-quality labels.

The ultimate goal of our work is to identify strategies for scalable and accurate medical data labeling. When crowd opinions differed from each other, the split in crowd votes was a useful predictor of the expert consensus label. Taken together with possible inherent ambiguity in some of the clips, this highlights a possible triage approach to data set labeling using gamified crowdsourcing. Crowd opinion for clips with a high degree of crowd agreement would be accepted as truth, and expert review would only be necessary for cases where crowd agreement drops below a certain threshold. This approach could significantly decrease the proportion of clips requiring expert review and optimize both the time and cost associated with current expert-based data set labeling approaches.

### Limitations

Our data set had an oversampling of clips with no lung pathology with more than 50% of clips in both the training and test data sets containing no B-lines. Since the crowd demonstrated higher concordance with reference standards than individual experts on clips with no B-lines but performed worse than experts on classifying discrete B-lines, it is possible that the crowd may be less efficient at identifying subtle diagnostic findings, but we are not adequately seeing this trend due to data set bias. While the similar or higher balanced multiclass concordance of the crowd compared with individual experts supports the conclusion that crowd performance was on par with experts, future work will apply more balanced data sets.

While this labeling iOS app is available to any user, users with medical backgrounds (medical students, etc) may be more likely to engage in this activity. We do have a general understanding of the proportion of our users with medical backgrounds, however, the precise demographic breakdown of prior medical experiences was poorly defined given that reports of demographic data from users were voluntary in the current platform. It is possible that our crowd is not representative of the general population and may be more consistent with a population of semiskilled labelers. The generalizability of our findings across variable crowd populations with clearly defined experience levels will need to be explored further. A breakdown of crowd performance by background medical expertise level will be helpful in future studies.

Our findings do show promise in streamlining the labeling of lung POCUS data; however, this work may not be generalizable to other more complex medical data labeling tasks. The next steps are to apply this approach to similar questions in lung POCUS data such as segmenting B-lines or evaluating for alternative findings beyond B-lines in lung POCUS. This will help us understand more about the generalizability of our labeling approach.

### Conclusions

We demonstrate that gamified crowdsourcing with strategic inbuilt quality control measures can produce B-line classification labels that match expert consensus better than individual experts themselves. These methods illustrate general strategies for improving the reliability of crowdsourced opinions on the task of classifying B-lines on lung POCUS clips. Using innovative and scalable approaches to generate high-quality labeled image databases could contribute to streamlining ML model development which could help either standardize or automate lung POCUS interpretation in the future. Future work can validate whether labels produced by crowdsourcing that achieve expert quality based on standard concordance metrics when used as training data, can produce models with comparable performance to those trained using expert-sourced training data.

## Appendix

Multimedia Appendix 1 Additional Methods.

## Abbreviations

ED: emergency department
ML: machine learning
POCUS: point-of-care ultrasound
Qscore: quality score

## Data Availability

The data sets analyzed during this study are available in the Harvard Dataverse repository [43]. Annotation data sets generated and analyzed during this study are available from the corresponding author on reasonable request.
