# Supplementary material for: Gamified Crowdsourcing as a Novel Approach to Lung Ultrasound Data Set Labeling: Prospective Analysis
Source: J Med Internet Res. 2024 Jul 4;26:e51397. doi: 10.2196/51397 (PMC11258523; doi:10.2196/51397)
Supplement: Multimedia Appendix 1 [file jmir_v26i1e51397_app1.docx]

**APPENDIX**

## **Gamified Crowdsourcing Contest Mechanics**

Users participating in classification contests were shown lung POCUS clips in random order. After submitting their opinion on a clip, they were either shown the correct label as feedback (on feedback clips) or a message thanking the user for their opinion (on non-feedback clips). Feedback clips consisted of the 195 training set clips with reference standard labels as well as any of the 1991 unlabeled clips that had acquired a crowd consensus label (Figure 1B). Feedback clips shown to the user were sampled to be equally likely to have a correct label in each of the three B-line classifications. Non-feedback clips included the 198 test clips with reference standard labels as well as any of the 1991 unlabeled clips that had yet to reach crowd consensus. Due to randomized clip ordering, users did not know which clips were feedback clips and therefore which clips would be used to grade their labeling performance for prizes. The live contest was run until all unlabeled clips reached crowd consensus.

### **Handling of Crowd Consensus Labels**

Crowd consensus (i.e., the B-line classification with the most crowd votes, with ties broken randomly) was achieved for an initially unlabeled clip when either (a) a minimum 3-vote difference between the highest and second highest-voted classification choice was achieved, or (b) 15 opinions were collected for the clip. If a user contributed multiple opinions to the same clip, only their most recent opinion was considered. Once crowd-consensus was reached on an unlabeled clip, the clip acquired a crowd consensus label and became a feedback clip.

###

### **Crowd User Quality Assessment**

A crowd user’s opinion quality score (Qscore) was defined as the user’s trailing average concordance (with respect to reference standards for training clips and crowd consensus labels for initially unlabeled clips) on the last 50 feedback clips they provided an opinion on. Each user’s Qscore was dynamically tracked over time and was updated with each opinion they gave. If a user’s Qscore was less than 80% at the time they gave an opinion on an individual clip, that opinion was not considered for determining the crowd consensus for that clip and instead was discarded. Users were assigned a Qscore of 0 until they provided at least 25 opinions on feedback clips. This ensured only the highest quality crowd opinions contributed to crowd consensus labels.

**Concordance Calculations**

For the single reference standard based on all six experts’ opinions, the average expert concordance with the reference standard was calculated as the average of the six individual expert concordances with the reference standard.

For concordances with respect to leave-one-out reference standards (based on all but one expert’s opinions), an individual expert’s concordance was the proportion of their opinions that matched their respective set of leave-one-out reference standard labels (the set of labels excluding their own opinion), and the crowd concordance was the proportion of the six leave-one-out reference standard labels matched by the crowd consensus label. The overall crowd concordance was the average of the per-clip crowd concordances.

**Concordance Statistical Testing**

For both tests of difference in concordance, each value in the paired sample consisted of the crowd consensus concordance and average expert concordance with the relevant reference standard(s) for a single test set clip. The differences obtained by subtracting the numbers in each pair were tested using a two-tailed one-sample Student’s *t*-test for difference from 0.

For the test comparing crowd and average individual expert concordance with the single reference standard created from all six experts’ opinions, the crowd consensus concordance for each clip was 1 if the crowd consensus label matched the reference standard and 0 otherwise, and the average expert concordance for that case was the proportion of the six experts’ opinions that matched the reference standard.

For the test comparing crowd and individual expert concordance with leave-one-out reference standards created from only five experts’ opinions each, the crowd consensus concordance for each clip was the proportion of the six leave-one-out reference standards that matched the crowd consensus label, and the average expert concordance for that clip was the proportion of the six experts that matched their respective leave-one-out reference standard (the one excluding their own opinion).
